# Association between post-traumatic stress disorder levels and serum inflammatory factors in patients undergoing digit replantation

**DOI:** 10.3389/fnins.2025.1719313

**Published:** 2026-01-16

**Authors:** Lei Ge, Hui Ju, Bing Liu, Chao Ma, Zhongrong Cheng, Panpan Cui, Wencong Liu

**Affiliations:** 1 Department of Emergency, People’s Hospital of Rizhao, Jining Medical University, Rizhao, Shandong, China; 2 Orthopedic Center, Rizhao Traditional Chinese Medicine Hospital, Rizhao, Shandong, China; 3 Nutrition Department, People’s Hospital of Rizhao, Jining Medical University, Rizhao, Shandong, China; 4 Department of ENT, People’s Hospital of Rizhao, Jining Medical University, Rizhao, Shandong, China; 5 College of Clinical Medicine, Jining Medical University, Jining, Shandong, China

**Keywords:** correlation analysis, digit replantation, inflammatory factors, influencing factors, post-traumatic stress disorder

## Abstract

**Objective:**

To investigate the levels of early postoperative post-traumatic stress disorder (PTSD) and their association with serum inflammatory factors in patients undergoing digit replantation, and to analyze the influencing factors.

**Methods:**

A total of 96 patients who underwent digit replantation at Rizhao People’s Hospital between March 2022 and December 2024 were enrolled 7 days postoperatively. PTSD levels were assessed using the PTSD Checklist-Civilian Version (PCL-C). Morning fasting blood samples were collected, and serum levels of interferon-γ (IFN-γ), tumor necrosis factor-α (TNF-α), interleukin-1β (IL-1β), IL-6, and IL-10 were measured by ELISA. Data were analyzed using univariate analysis, Spearman’s correlation, multiple linear regression, and Receiver Operating Characteristic (ROC) curve analysis.

**Results:**

The mean PCL-C score for the 96 patients was 38.21 ± 9.31, with 44 patients (45.83%) presenting PTSD symptoms. Univariate analysis revealed that gender, education level, injury type, complete amputation, involvement of the dominant hand, and number of amputated digits significantly influenced PCL-C scores (*p* < 0.05). PCL-C scores showed positive correlations with both anxiety and depression scores (*r* = 0.285 and 0.679, respectively, *p* < 0.01). Multiple linear regression identified gender, education level, complete amputation, number of injured digits, and levels of anxiety and depression as independent influencing factors for PTSD (*p* < 0.05). Correlation analysis indicated that PCL-C scores were positively correlated with IFN-γ, TNF-α, IL-1β, and IL-6 levels (*r* = 0.581, 0.521, 0.552, and 0.507, respectively), and negatively correlated with IL-10 (*r* = −0.474, *p* < 0.01). ROC curve analysis suggested that serum inflammatory factors have good predictive value for PTSD.

**Conclusion:**

Patients exhibit a certain degree of PTSD in the early stage after digit replantation. Its occurrence is closely associated with female gender, lower education level, severity of the trauma, and co-morbid anxiety and depression, and is significantly correlated with an imbalance between pro-inflammatory and anti-inflammatory serum factors. Serum inflammatory factors may serve as potential biological markers for the early identification of PTSD risk.

## 1 Introduction

Hand trauma is one of the most common conditions encountered in orthopedics, characterized by high disability rates and poor prognoses, often leading to varying degrees of hand dysfunction in patients (Mock and Cherian, 2008). Among these injuries, digit amputation represents a severe form of limb trauma. It not only causes significant physical functional impairment but also inflicts profound psychological distress, predisposing patients to post-traumatic psychological disorders (Maddison et al., 2023; Ladds et al., 2017). Post-traumatic Stress Disorder (PTSD) is a persistent mental disorder that occurs after an individual experiences or witnesses a traumatic event. Studies have shown that patients frequently experience significant emotional problems, such as anxiety and depression, following digit replantation surgery (Yehuda, 2002). Furthermore, foreign follow-up surveys indicate that nearly 50% of patients with hand trauma may develop PTSD postoperatively (Weis et al., 2012). PTSD severely impacts patients’ physical and psychological rehabilitation, familial economics, and social interactions, and can lead to social withdrawal, anger, and aggressive behaviors. Therefore, early assessment and intervention for PTSD in patients after digit replantation are crucial.

Historically, research on the pathological mechanisms of PTSD has primarily focused on neuroanatomical and psychological cognitive domains. However, the “immune-inflammation” hypothesis has recently gained increasing attention for its role in the pathogenesis of mental disorders (Smith, 1991). This hypothesis posits that major traumatic stress can activate the body’s immune system, leading to increased release of pro-inflammatory cytokines (such as tumor necrosis factor-α, interleukin-1β, interleukin-6, etc.) and a relative insufficiency of anti-inflammatory cytokines (such as IL-10). This imbalance in the inflammatory state may directly contribute to the onset and progression of PTSD by affecting neuroendocrine functions, neurotransmitter metabolism, and neuroplasticity (Tan et al., 2023; Elgellaie et al., 2023; Guo et al., 2023). Research indicates that levels of pro-inflammatory cytokines like IL-6, TNF-α are significantly elevated in the peripheral blood of PTSD patients and correlate with symptom severity (Toft et al., 2022; Hori and Kim, 2019). This suggests that serum inflammatory factors might serve as a biological bridge connecting physical trauma to psychological trauma.

Although existing studies have separately explored the epidemiology of PTSD in digit replantation patients and changes in inflammatory factors in patients with mental disorders, research combining these two aspects remains scarce. Direct evidence elucidating the intrinsic relationship between PTSD levels and specific serum inflammatory factors (such as IFN-γ, TNF-α, IL-1β, IL-6, IL-10) in this specific trauma population is still lacking. This gap limits our in-depth understanding of the pathophysiological mechanisms of PTSD following digit replantation from a biomedical perspective and hinders the development of biomarker-based early identification and intervention strategies for this population.

Therefore, this study aims to fill this research gap. We intend to enroll patients who have undergone digit replantation, assess their PTSD levels, and concurrently measure the serum concentrations of key inflammatory factors, including IFN-γ, TNF-α, IL-1β, IL-6, and IL-10. Through correlation analysis, we will thoroughly investigate the relationship between the severity of PTSD and the levels of specific inflammatory factors. This study not only holds the potential to reveal a novel mechanism for PTSD development in digit replantation patients via the “inflammation-psychology” axis but may also provide a crucial theoretical and laboratory basis for the clinical early screening of high-risk PTSD patients and the future development of targeted anti-inflammatory adjuvant therapy strategies, possessing significant clinical relevance and scientific value.

## 2 Materials and methods

### 2.1 Study subjects

#### 2.1.1 Sample size calculation

This study is a cross-sectional observational study, and the sample size was determined based on the statistical requirements of multiple linear regression analysis and correlation analysis. Referring to statistical standards, each independent variable in multiple linear regression requires matching 10–20 samples to ensure statistical power. Combined with the result that the incidence of PTSD in hand trauma patients reported in previous studies is 40–50% (Maddison et al., 2023; Weis et al., 2012; Tolin and Foa, 2006), *α* = 0.05 and *β* = 0.10 (statistical power of 80%) were set. A total of 8 core independent variables were included in this study. Calculated at 10 samples per independent variable, a minimum of 80 samples were required. Considering a potential attrition rate of 20%, 96 patients were planned to be enrolled.

#### 2.1.2 Sampling method

Consecutive sampling was adopted in this study. From March 2022 to December 2024, all patients who met the inclusion criteria and were 7 days postoperatively after digit replantation in the Department of Hand and Foot Surgery of Rizhao People’s Hospital were consecutively enrolled in the order of admission to ensure sample representativeness.

#### 2.1.3 Inclusion and exclusion criteria

Inclusion criteria: Patients who underwent digit replantation and survived 7 days postoperatively; those with clear consciousness who could read and correctly answer questions; age >18 years old. Exclusion criteria: Patients with major diseases such as malignant tumors and cardiovascular diseases; those with other major traumatic events; those with mental abnormalities who could not cooperate.

All participants underwent digit replantation within 8 h after injury. Researchers conducted assessments and serological tests in the early morning on the 7th day postoperatively (7:00–9:00 a.m.). This study was approved by the Ethics Committee of Rizhao People’s Hospital (approval number: MR-95-03) and registered in the Chinese Clinical Trial Registry. All participants signed the informed consent form.

### 2.2 General data

Collected data included patient gender, age, marital status, economic income, education level, smoking history, alcohol use, and body mass index (BMI). Disease-related data included the type of injury, whether the thumb was injured, whether the amputation was complete, whether the dominant hand was injured, and the number of injured digits.

### 2.3 Research methods

#### 2.3.1 Assessment of PTSD levels

The PTSD Checklist-Civilian Version (PCL-C) was used to assess PTSD levels, covering three dimensions: re-experiencing, avoidance/numbing, and hyperarousal. A total score of 17–37 indicates no significant PTSD symptoms, 38–49 suggests partial symptoms, and 50–85 indicates significant symptoms (Mureșanu et al., 2022). Participants with a PCL-C total score <38 were classified into the no-PTSD-symptoms group, and those with a score ≥38 were classified into the PTSD-symptoms group.

#### 2.3.2 Assessment of anxiety and depression levels

The Self-Rating Anxiety Scale (SAS) was used to assess anxiety levels, with a score equal to or exceeding 50 indicating the presence of anxiety (Dunstan and Scott, 2020). The Self-Rating Depression Scale (SDS) was used to assess depression levels, with a score of 53 or above indicating depressive symptoms (Jokelainen et al., 2019). The SAS and SDS are internationally recognized scales that can be administered by non-professionals. They are widely used for personal psychological care and mental health status monitoring. Furthermore, they are the most extensively used tools for assessing anxiety and depression, demonstrating good reliability and validity.

#### 2.3.3 Detection of serum indicators

All blood samples were collected in the morning (between 7:00 a.m. and 9:00 a.m.) after an overnight fast. Samples were immediately transported to the central laboratory of our hospital for the detection of serum inflammatory factors, including interferon-γ (IFN-γ), tumor necrosis factor-α (TNF-α), interleukin-1β (IL-1β), IL-6, and IL-10. All inflammatory factors were measured using enzyme-linked immunosorbent assay (ELISA). All kits were provided by Shanghai Enzyme-linked Biotechnology Co., Ltd.

#### 2.3.4 Control of potential confounding factors

##### 2.3.4.1 Data quality control

There was no missing data in this study (missing rate = 0%). Scale data and serum test results were double-checked by two researchers to ensure data integrity and accuracy.

##### 2.3.4.2 Postoperative pain control

All patients received standardized analgesic treatment to avoid the interference of differences in pain intensity on inflammatory factor release and psychological status.

##### 2.3.4.3 Consistency of surgical trauma

All patients underwent digit replantation within 8 h after injury, with unified surgical methods and anesthesia plans to reduce confounding caused by differences in the degree and timing of surgical trauma.

##### 2.3.4.4 Infection exclusion

The inclusion criteria were limited to “patients who survived 7 days after surgery.” Within 7 days postoperatively, daily monitoring of wound redness, swelling, exudation, and body temperature changes was performed, and patients with concurrent infections were excluded to avoid interference of infection related inflammatory responses on the test results.

#### 2.3.5 Data analysis

SPSS 25.0 was used for statistical analysis of the data. The Shapiro–Wilk test was used to verify the normality of quantitative variables (PCL-C score, SAS score, SDS score, and serum levels of various inflammatory factors). Normally distributed data were expressed as mean ± standard deviation, while non-normally distributed data were presented as median and interquartile range. Qualitative data were described as frequencies and percentages. The Mann–Whitney U test was used for two-group comparisons, one-way analysis of variance (ANOVA) was used for multi-group comparisons, Spearman’s correlation analysis was employed for assessing relationships, and multiple linear regression analysis was used to screen for influencing factors. The diagnostic value of serum inflammatory factor levels for PTSD in digit replantation patients was analyzed using Receiver Operating Characteristic (ROC) curves. Statistical significance was set at *p* < 0.05.

## 3 Results

### 3.1 Clinical characteristics of the study subjects

A total of 96 patients who underwent digit replantation were enrolled, with a mean age of 39.85 ± 7.36 years. The largest proportion (45.83%) was in the 40–49 age group. Males constituted the majority (71.88%). Most patients resided in rural areas (79.17%). Regarding marital status, the vast majority were married (78.13%). Education level was predominantly junior high school (52.08%). The mean monthly household income per capita was 7227.08 ± 1620.29 RMB, with 81.25% of patients having an income between 5,000 and 10,000 RMB. A history of smoking and alcohol use was reported in 69.79 and 78.13% of patients, respectively. The mean BMI for all patients was 23.90 ± 2.578 kg/m^2^, with 54.17% falling within the normal weight range. Regarding injury characteristics, laceration was the most common type (43.75%). The thumb was involved in 66.67% of cases. Incomplete amputation was predominant (54.17%), and the dominant hand was injured in 66.67% of patients. The number of amputated digits was primarily 1–2 (54.17%). Details are presented in Table 1.

**Table 1:** Clinical characteristics of patients undergoing digit replantation.

Characteristic	Category	*n*	%
Gender	Male	69	71.88%
	Female	27	28.13%
Age (years)	18–29	10	10.42%
	30–39	33	34.38%
	40–49	44	45.83%
	>50	9	9.38%
Residence	Urban	20	20.83%
	Rural	76	79.17%
Marital status	Unmarried	12	12.50%
	Married	75	78.13%
	Divorced	5	5.21%
	Widowed	4	4.17%
Education level	Primary or below	14	14.58%
	Junior high school	50	52.08%
	High school or above	32	33.33%
Monthly income (RMB)	<5,000	13	13.54%
	5000–10000	78	81.25%
	>10,000	5	5.21%
Smoking history	No	29	30.21%
	Yes	67	69.79%
Alcohol use	No	21	21.88%
	Yes	75	78.13%
BMI (kg/m2)	Underweight (<18.5)	9	9.38%
	Normal (18.5–23.9)	52	54.17%
	Obese (≥28.0)	19	19.79%
	Overweight (24.0–27.9)	16	16.67%
Injury type	Crush	31	32.29%
	Laceration	42	43.75%
	Avulsion	21	21.88%
	Other	2	2.08%
Thumb involvement	No	32	33.33%
	Yes	64	66.67%
Complete amputation	No	52	54.17%
	Yes	44	45.83%
Dominant hand injured	No	32	33.33%
	Yes	64	66.67%
Number of amputated digits	1–2	52	54.17%
	3–4	28	29.17%
	>5	16	16.67%

### 3.2 PTSD levels in patients after digit replantation

After the Shapiro–Wilk test, the PCL-C scores of 96 patients who underwent digit replantation were normally distributed 7 days postoperatively. The mean PCL-C total score for the 96 patients on the seventh postoperative day was 38.21 ± 9.31. According to the PCL-C scale classification, this indicates a mild level of PTSD symptoms. Fifty-two patients (54.17%) showed no PTSD symptoms, while 44 patients (45.83%) exhibited PTSD symptoms.

### 3.3 Analysis of factors influencing PTSD levels after digit replantation

#### 3.3.1 Univariate analysis of PTSD levels by clinical characteristics

Analysis of differences in PCL-C scores across different clinical characteristics revealed significant associations (*p* < 0.05) with gender, education level, injury type, involvement of the thumb, complete amputation, injury to the dominant hand, and the number of amputated digits. Details are shown in Table 2.

**Table 2:** Univariate analysis of factors associated with PTSD levels in patients undergoing digit replantation.

Variable	Category	*n*	PCL-C score (mean ± SD)	*F*/*t* value	*p* value
Gender	Male	69	36.62 ± 9.42	7.613	0.007
	Female	27	42.26 ± 7.789		
Education level	Primary or below	14	33.07 ± 8.766	3.883	0.024
	Junior High School	50	40.34 ± 9.273		
	High School or above	32	37.13 ± 8.765		
Injury type	Crush	31	32.52 ± 7.159	20.746	< 0.001
	Laceration	42	37.12 ± 6.153		
	Avulsion	21	48.57 ± 8.936		
	Other	2	40.5 ± 14.849		
Thumb involvement	No	32	32.44 ± 7.057	22.666	< 0.001
	Yes	64	41.09 ± 8.984		
Complete amputation	No	52	32.06 ± 5.589	102.508	< 0.001
	Yes	44	45.48 ± 7.382		
Dominant hand injured	No	32	32.44 ± 7.057	22.666	< 0.001
	Yes	64	41.09 ± 8.984		
Number of amputated digits	1–2	52	31.92 ± 5.401	49.324	< 0.001
	3–4	28	44 ± 4.119		
	>5	16	51.33 ± 7.808		

#### 3.3.2 Correlation between PTSD levels and anxiety/depression after digit replantation

The mean SAS score was 53.96 ± 4.72, with 75% of patients meeting the criteria for anxiety. The mean SDS score was 56.32 ± 5.05, with 63.54% of patients meeting the criteria for depression. Spearman’s correlation analysis was used to explore the relationships between PTSD symptoms and anxiety/depression. The results indicated significant positive correlations between PTSD symptoms and both anxiety and depression scores. Details are presented in Table 3.

**Table 3:** Correlation analysis of PTSD, anxiety, and depression scores in patients undergoing digit replantation.

Variable	PCL-C	SAS	SDS
PCL-C	1		
SAS	0.285**	1	
SDS	0.679**	0.416**	1

**p < 0.01.

#### 3.3.3 Multiple linear regression analysis of PTSD levels after digit replantation

Based on the results of the univariate and correlation analyses, gender, education level, injury type, involvement of the thumb, complete amputation, injury to the dominant hand, number of injured digits, anxiety score, and depression score were included as independent variables, with PTSD symptoms as the dependent variable in a multiple linear regression model. The Variance Inflation Factor (VIF) for all variables was less than 10, indicating no severe multicollinearity. The analysis revealed that gender, education level, complete amputation, number of injured digits, anxiety level, and depression level were significant independent factors influencing PTSD levels (*p* < 0.05). Variable assignments are shown in Table 4, and the regression results are presented in Table 5.

**Table 4:** Variable assignment for regression analysis.

Variable	Assignment
Gender	Male = 0; female = 1
Education level	Primary or below = (0, 0); junior high school = (1, 0); high school or above = (0, 1) (dummy variables)
Injury type	Crush = (0, 0, 0); laceration = (1, 0, 0); avulsion = (0, 1, 0); other = (0, 0, 1) (dummy variables)
Thumb involvement	No = 0; yes = 1
Complete amputation	No = 0; yes = 1
Dominant hand injured	No = 0; yes = 1
Number of amputated digits	1–2 = (0, 0); 3–4 = (1, 0); >5 = (0, 1)
Anxiety level (SAS)	Entered as the original value
Depression level (SDS)	Entered as the original value

**Table 5:** Multiple linear regression analysis of factors associated with PTSD symptoms.

Variable	Category	Unstandardized *β*	SE	Standardized *β*	*t*-value	*p*-value
Constant		−25.544	7.669		−3.331	0.001
Gender	Male					
	Female	2.208	1.059	0.107	2.085	0.04
Education level	Primary or below					
	Junior high school	3.48	1.383	0.188	2.517	0.014
	High school or above	2.426	1.402	0.124	1.731	0.087
Complete Amputation	No					
	Yes	4.906	2.404	0.264	2.041	0.044
Number of amputated digits	1–2					
	3–4	5.798	2.221	0.285	2.61	0.011
	>5	8.946	3.155	0.32	2.835	0.006
Anxiety level (SAS)		0.083	0.107	0.042	1.97	0.043
Depression level (SDS)		0.323	0.129	0.175	2.5	0.014

*R*^2^ = 0.83, Adjusted *R*^2^ = 0.800; *F* = 28.17, *p* < 0.001.

### 3.4 Correlation between PTSD levels and serum inflammatory factors after digit replantation

#### 3.4.1 Correlation analysis in all patients

Spearman’s correlation analysis conducted on all patients (*n* = 96) showed that PTSD levels were significantly correlated with serum levels of IFN-γ, TNF-α, IL-1β, IL-6, and IL-10 (*p* < 0.05). PTSD levels were positively correlated with IFN-γ (*r* = 0.581), TNF-α (*r* = 0.521), IL-1β (*r* = 0.552), and IL-6 (*r* = 0.507), and negatively correlated with IL-10 (*r* = −0.474). Details are shown in Table 6.

**Table 6:** Correlation analysis between PTSD levels and serum inflammatory factors in all patients undergoing digit replantation.

Variable	PCL-C	IFN-γ	TNF-α	IL-1β	IL-6	IL-10
PCL-C	1					
IFN-γ	0.581**	1				
TNF-α	0.521**	0.747**	1			
IL-1β	0.552**	0.736**	0.806**	1		
IL-6	0.507**	0.782**	0.723**	0.700**	1	
IL-10	−0.474**	−0.684**	−0.615**	−0.556**	−0.724**	1

**p < 0.01.

#### 3.4.2 Comparison of serum inflammatory factors between no-PTSD-symptoms and PTSD-symptoms groups

Significant differences were observed in the serum levels of IFN-γ, TNF-α, IL-1β, IL-6, and IL-10 between the no-PTSD-symptoms group and the PTSD-symptoms group. In the no-PTSD-symptoms group, the levels were: IFN-γ: 12.19 ± 2.04 pg/mL, TNF-α: 51.08 ± 4.36 ng/L, IL-1β: 44.10 ± 4.34 ng/L, IL-6: 60.37 ± 4.32 ng/L, IL-10: 13.07 ± 1.93 ng/L. In the PTSD-symptoms group, the levels were: IFN-γ: 14.92 ± 2.14 pg/mL, TNF-α: 56.44 ± 4.57 ng/L, IL-1β: 49.88 ± 4.36 ng/L, IL-6: 65.41 ± 4.45 ng/L, IL-10: 11.47 ± 1.55 ng/L. The differences in all these parameters between the two groups were statistically significant (*p* < 0.001) (see Table 7).

**Table 7:** Comparison of serum inflammatory factor levels between No-PTSD-symptoms and PTSD-symptoms groups.

Group	IFN-γ (pg mL−1)	TNF-α (ng/L)	IL-1β (ng/L)	IL-6 (ng/L)	IL-10 (ng/L)
No-PTSD-symptoms	12.19 ± 2.04	51.08 ± 4.36	44.10 ± 4.34	60.37 ± 4.32	13.07 ± 1.93
PTSD-symptoms	14.92 ± 2.14	56.44 ± 4.57	49.88 ± 4.36	65.41 ± 4.45	11.47 ± 1.55
*t*-value	−6.39	−5.863	−6.485	−5.611	4.403
*p*-value	*p* < 0.001	*p* < 0.001	*p* < 0.001	*p* < 0.001	*p* < 0.001

Within the PTSD-symptoms group, PTSD levels showed significant correlations with IFN-γ (*r* = 0.427, *p* < 0.01), TNF-α (*r* = 0.426, *p* < 0.01), IL-1β (*r* = 0.428, *p* < 0.01), IL-6 (*r* = 0.389, *p* < 0.01), and IL-10 (*r* = −0.458, *p* < 0.01). In contrast, no significant correlations were found between PTSD levels and any of the inflammatory factors in the no-PTSD-symptoms group (*p* > 0.05) (see Tables 8, 9).

**Table 8:** Correlation analysis between PTSD levels and serum inflammatory factors within the PTSD-symptoms group.

Variable	IFN-γ	TNF-α	IL-1β	IL-6	IL-10	PCL-C
IFN-γ	1					
TNF-α	0.743**	1				
IL-1β	0.806**	0.929**	1			
IL-6	0.707**	0.819**	0.900**	1		
IL-10	−0.714**	−0.812**	−0.894**	−0.850**	1	
PCL-C	0.427**	0.426**	0.428**	0.389**	−0.458**	1

**p < 0.01.

**Table 9:** Correlation analysis between PTSD levels and serum inflammatory factors within the No-PTSD-symptoms group.

Variable	IFN-γ	TNF-α	IL-1β	IL-6	IL-10	PCL-C
IFN-γ	1					
TNF-α	0.557**	1				
IL-1β	0.454*	0.551**	1			
IL-6	0.395*	0.452**	0.312*	1		
IL-10	−0.528*	−0.312*	−0.12	−0.530**	1	
PCL-C	−0.02	−0.236	−0.233	−0.178	−0.07	1

**p < 0.01; *p < 0.05.

### 3.5 ROC curve analysis

**Figure 1:**
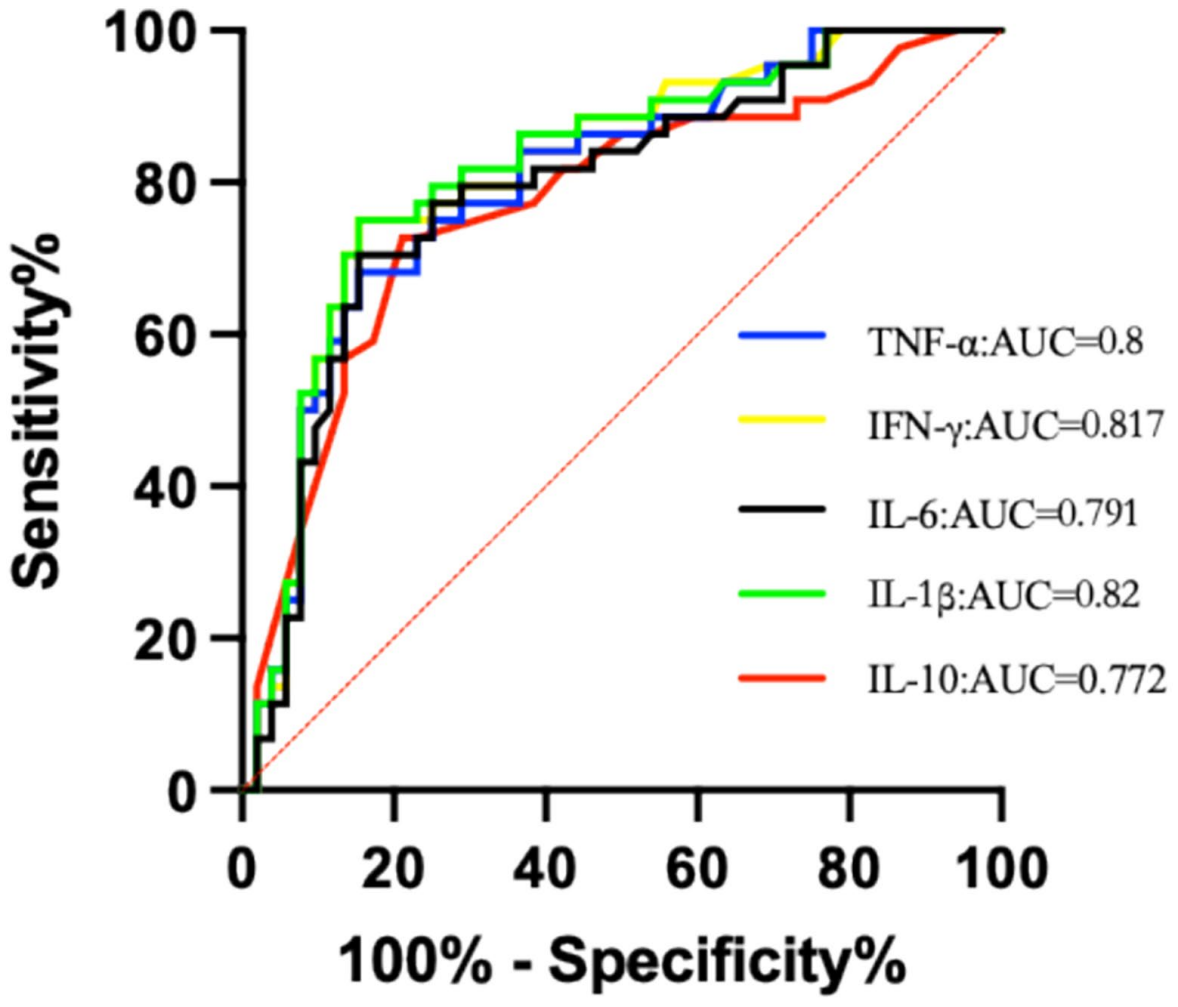
ROC curves of serum inflammatory factors for diagnosing PTSD.

ROC curve analysis indicated that for predicting PTSD symptoms in digit replantation patients, the optimal cut-off value for IFN-γ was 13.95 pg/mL, with an AUC of 0.817 (95% CI: 0.73–0.904), sensitivity of 75%, and specificity of 84.6%. For TNF-α, the cut-off was 54.9 ng/L, AUC = 0.8 (95% CI: 0.71–0.89), sensitivity = 68.2%, specificity = 83.1%. For IL-1β, the cut-off was 48.5 ng/L, AUC = 0.82 (95% CI: 0.733–0.907), sensitivity = 70.5%, specificity = 86.5%. For IL-6, the cut-off was 63.7 ng/L, AUC = 0.791 (95% CI: 0.698–0.884), sensitivity = 70.5%, specificity = 80.8%. For IL-10, the cut-off was 11.4 ng/L, AUC = 0.772 (95% CI: 0.675–0.869), sensitivity = 72.7%, specificity = 78.8%. After grouping participants based on these cut-off values, the chi-square test showed significant differences in the distribution of PTSD outcomes between the groups (*p* < 0.001) (see Figure 1, Tables 10, 11).

**Table 10:** Cut-off values of serum inflammatory factors for diagnosing PTSD.

Inflammatory factor	AUC	95% CI lower	95% CI upper	*p*-value	Cut-off	Sensitivity	Specificity
IFN-γ	0.817	0.73	0.904	*p* < 0.001	13.95	75%	84.6%
TNF-α	0.8	0.71	0.89	*p* < 0.001	54.9	68.2%	83.1%
IL-1β	0.82	0.733	0.907	*p* < 0.001	48.5	70.5%	86.5%
IL-6	0.791	0.698	0.884	*p* < 0.001	63.7	70.5%	80.8%
IL-10	0.772	0.675	0.869	*p* < 0.001	11.4	72.7%	78.8%

**Table 11:** Comparison of PTSD incidence stratified by inflammatory factor cut-off values.

Group	IFN-γ		TNF-α		IL-1β		IL-6		IL-10	
Cut-off	<13.95	≥13.95	<54.9	≥54.9	<48.5	≥48.5	<63.7	≥63.7	<11.4	≥11.4
No-PTSD-symptoms	43	9	44	8	45	7	42	10	11	41
PTSD-symptoms	11	33	14	30	13	31	13	31	32	12
*χ*^2^ Value	32.2344		27.78		32.37		25.56		25.64	
*p*-value	*p* < 0.001		*p* < 0.001		*p* < 0.001		*p* < 0.001		*p* < 0.001	

## 4 Discussion

### 4.1 PTSD at a mild level in patients following digit replantation

In this study, assessments and serological examinations were performed on the early morning of the 7th day after surgery (7:00–9:00 a.m.), mainly based on the following considerations: The 1–3 days after digit replantation represent the peak period of acute post-traumatic inflammatory response, which is significantly affected by acute stressors such as surgical trauma, pain, and edema. By the 7th day postoperatively, the patient’s wound healing enters a stable phase, and the acute inflammatory response gradually subsides. At this time, the detected inflammatory factor levels can better reflect the chronic inflammatory state associated with psychological stress, rather than the interference from acute inflammation caused solely by physical trauma (Broadbent et al., 2003; Ge et al., 2025). Early symptoms of PTSD usually begin to manifest around 1 week after trauma; by the 7th day postoperatively, patients have initially adapted to the traumatic event and postoperative state, enabling them to complete psychological scale assessments more accurately and avoiding scale response bias caused by physical distress in the early postoperative period (1–3 days) (Broadbent et al., 2003; Glaser and Kiecolt-Glaser, 2005). Additionally, the 7th day postoperatively is a key node for the first rehabilitation assessment of patients undergoing digit replantation. At this time, patients are still during hospitalization, facilitating the unified collection of fasting blood samples and completion of centralized assessments, thus ensuring the standardization and integrity of data collection.

In this cohort of 96 digit replantation patients, the mean PCL-C total score on postoperative day 7 was 38.21 ± 9.31. According to the PCL-C scale classification, this indicates a mild level of post-traumatic stress disorder symptoms. Fifty-two patients (54.17%) exhibited no PTSD symptoms, while 44 (45.83%) presented with PTSD symptoms. The PTSD level observed in our study was higher than that reported in a survey of 1,386 accidental trauma patients by Alarcon et al. (2012). This discrepancy may be attributed to differences in the nature of the injuries. The cohort investigated by Alarcon consisted of patients from a trauma clinic, whose injuries were likely less severe than digit amputations, although that study also noted a positive correlation between PTSD levels and injury severity. Digit replantation patients typically sustain injuries such as lacerations or crush injuries to the hand. The success rate of replantation is considerably influenced by psychological factors; patient stress and anxiety can easily induce vasoconstriction and vascular crisis, leading to replantation failure, finger necrosis, and permanent physical disability. Yu et al. (2023) reported that the psychological state of digit replantation patients is a significant factor affecting surgical survival rates, indicating the presence of post-traumatic psychological stress disorders and their substantial impact on postoperative outcomes. During our investigation, researchers also observed that patients who suffered digit amputation experienced profound psychological trauma from the sudden accident, frequently re-experiencing the traumatic scene in their minds and even in dreams, severely affecting their psychological and emotional state. Being psychologically unable to accept the reality, patients often adopt negative avoidance attitudes and fail to cooperate with treatment. After experiencing the painful trauma, patients remain hypervigilant towards their surroundings, worrying about poor postoperative functional recovery of the limb and decreased quality of life. Healthcare professionals should provide effective psychological counseling tailored to individual patient personalities, guide patients in actively seeking solutions, encourage them to choose positive and effective coping strategies, and help reduce PTSD levels in digit replantation patients.

### 4.2 Influencing factors of PTSD in patients following digit replantation

The results of the univariate analysis in this study showed a statistically significant difference in PCL-C total scores between genders, with female patients scoring 42.26 ± 7.789, higher than male patients (*p* < 0.05). This suggests a more pronounced tendency towards PTSD in females, consistent with the findings of Ranasinghe et al. (2022). Previous research has reported female gender as a risk factor for developing PTSD (Polimanti et al., 2017). A review by Tolin and Foa (2006) of gender differences in stress disorders over the past 25 years indicated that women are more susceptible to PTSD and that female gender is a risk factor for the disorder. This may be related to different biological and genetic substrates in females and males. Women tend to be more perceptive, emotionally nuanced, and emotionally reactive, with potentially lower psychological resilience compared to men, leading to a more profound experience following a traumatic event. Therefore, in addition to necessary diagnostic and therapeutic procedures and psychological comfort, clinical staff can utilize self-help mindfulness interventions to assist patients in alleviating stress. A UK meta-analysis studying the effects of mindfulness- or acceptance-based self-help psychological interventions found that self-help mindfulness interventions can alleviate negative emotions, offer good cost-effectiveness, and can be promoted and implemented in routine clinical care (Cavanagh et al., 2014).

In this study, PCL-C total scores also differed significantly among patients with different education levels (*p* < 0.01). Numerous analyses of factors influencing PTSD have generally concluded an inverse correlation between PTSD symptoms and education level (Lee et al., 2015). The reasons for this may be that individuals with higher education have a greater capacity for acquiring knowledge, a more accurate understanding of the disease and its outcomes, and easier access to methods for coping with stressful events, leading them to adopt more positive cognitive and coping styles. Those with lower education levels are more prone to negative cognitions when facing accidental injuries, resulting in a higher incidence of PTSD. Therefore, during the hospitalization of digit replantation patients, for those with lower educational attainment, healthcare workers should develop corresponding health education plans according to the patient’s literacy level, effectively conduct education regarding disease treatment and preventive healthcare, help patients understand the disease progression and potential challenges, eliminate psychological burdens early, and assist in alleviating psychological pressure.

The results of this study also indicate a close relationship between the severity of the hand injury and PTSD levels, particularly in patients with complete amputations and multiple digit amputations. This aligns with the findings of Ghițan et al. (2023). Firstly, complete and multiple digit amputations represent highly impactful physical trauma events. The accompanying intense pain, significant bleeding, and the sight of tissue destruction constitute a powerful, overwhelming negative psychological experience. This experience easily forms vivid and strong traumatic memories in the patient’s mind, manifesting as core PTSD symptoms, such as uncontrollable recurrent recollections of the accident scene or nightmares. Secondly, the fine motor function of the fingers is central to an individual’s occupational ability and daily self-care. Complete and multiple digit amputations imply a potentially worse functional prognosis. The profound anxiety patients develop regarding returning to work, maintaining livelihood, and self-care abilities becomes a persistent psychological stressor, continuously eroding the foundation of their psychological recovery, leading to persistent negative emotional states and hypervigilance (Dhanaraj et al., 2024; Meyer, 2003). The prolonged and painful rehabilitation cycle for digit replantation patients, including repeated functional exercises and potential multiple revision surgeries, constantly reminds the patient of the initial traumatic event throughout the treatment period. This process not only consumes the patient’s physical endurance but also poses a continuous test to their psychological resilience, thereby maintaining and exacerbating various PTSD symptoms (Pinto et al., 2016; Siqveland et al., 2017). Consequently, in clinical practice, for patients with such high trauma severity, psychological assessment and early warning should be initiated early, and timely, systematic psychological intervention should be provided throughout the entire treatment and rehabilitation cycle to improve their long-term physical and mental rehabilitation outcomes.

The results showed that the mean SAS score in post-replantation patients was 53.96 ± 4.72, with 75% of patients meeting criteria for anxiety. The mean SDS score was 56.32 ± 5.05, with 63.54% of patients meeting criteria for depression. Furthermore, significant positive correlations were found between PTSD symptoms and both anxiety and depression scores after replantation. This is consistent with the results of Roth et al. (2023). Individuals exhibit significant emotional reactions following traumatic events. Patients with anxiety and depression are more inclined to interpret events around them as malignant and negative, displaying a pessimistic expectation state, thereby increasing their susceptibility to PTSD. Therefore, while focusing on PTSD symptoms, anxiety and depressive emotions should not be overlooked. Research has found that anxiety and depression easily lead to a state of hypervigilance, indirectly exacerbating the occurrence of PTSD. It is inferred that in digit replantation patients, anxiety and depression may indirectly influence PTSD as mediating variables. Further analysis of the interaction mechanisms among multiple variables with an expanded sample size is warranted. It is recommended that during patient treatment, close attention be paid to changes in the psychological state of patients after multiple digit replantation, aiming to reduce patient anxiety and depression, thereby lowering the incidence of PTSD.

### 4.3 Analysis of the correlation between serum inflammatory factors and PTSD levels in digit replantation patients

The strong positive correlations observed in this study between PTSD symptoms and multiple pro-inflammatory factors (IFN-γ, TNF-α, IL-1β, IL-6) are highly consistent with the immune-inflammation hypothesis concerning psychological disorders following physical trauma (Peruzzolo et al., 2022; Kim et al., 2016). It should be noted that factors such as surgical trauma and postoperative pain following digit replantation can themselves activate the hypothalamic–pituitary–adrenal (HPA) axis and the sympathetic nervous system (SNS), leading to a transient increase in pro-inflammatory factors (Black, 2002; Steptoe et al., 2007; Yadav et al., 2009; Lee et al., 2022), which constitute potential confounding factors in this study. However, the following results in this study support the specific association between inflammatory factors and PTSD, rather than the impact of simple traumatic stress: Firstly, the correlation between inflammatory factors and PTSD only exists within the PTSD symptom group, with no significant association observed in the non-PTSD group, suggesting that simple acute traumatic stress is insufficient to maintain the persistent correlation between inflammatory factors and psychological symptoms. Secondly, the anti-inflammatory factor IL-10 is significantly decreased in the PTSD symptom group, presenting the imbalanced characteristic of “increased pro-inflammatory factors and decreased anti-inflammatory factors” rather than a single elevation of pro-inflammatory factors. This is different from the manifestation of acute inflammatory response after simple trauma. Thirdly, after controlling for confounding factors through standardized analgesia, infection exclusion, and multivariate regression models, the correlation between inflammatory factors and PTSD remains statistically significant, further weakening the interference of confounding factors.

We hypothesize the existence of a “trauma-stress-neuroinflammation” vicious cycle: the initial traumatic event triggers immune activation; in susceptible individuals, this inflammatory state may affect the central nervous system by crossing the blood–brain barrier, for instance, by enhancing fear responses in the amygdala, impairing emotional regulation functions of the prefrontal cortex, and disrupting memory integration in the hippocampus, thereby promoting the onset and development of PTSD (Yu et al., 2022; Starcevic et al., 2014). Conversely, the chronic psychological stress state inherent to PTSD itself (persistent alertness, recurrent intrusive memories) can, in turn, persistently activate the SNS, stimulating immune cells to further release inflammatory factors, plunging the body into a chronic, low-grade inflammatory state (Wang et al., 2022). Simultaneously, the anti-inflammatory factor IL-10 was significantly lower in the PTSD-symptoms group and negatively correlated with PTSD scores. This aligns with the theoretical framework of the “Inflammatory Response System (IRS) and Compensatory Immune-Regulatory Reflex System (CIRS)” proposed by Ferencova et al. (2022). Under healthy conditions, after the initiation of a pro-inflammatory response, the CIRS (IL-10) responds rapidly to suppress inflammation and restore immune homeostasis. Our results indicate that in PTSD patients, not only is the IRS (represented by IFN-γ, TNF-α, IL-1β, IL-6) activated, but the function of the CIRS (represented by IL-10) may be relatively insufficient or depleted. This disruption of the “pro-inflammatory/anti-inflammatory” balance, rather than the elevation of individual pro-inflammatory factors alone, might be a more core immunological feature of PTSD. The decrease in IL-10 implies that the body loses a key mechanism to effectively curb the spread of inflammation, which may partially explain why the inflammatory state persists in PTSD patients and is closely linked to their symptom severity.

This study found that serum inflammatory factors IFN-γ and IL-1β demonstrated the strongest correlations with PTSD (*r* = 0.581 and 0.552, respectively) and exhibited excellent predictive performance (AUC = 0.817 and 0.820, respectively). IFN-γ, as a potent pro-inflammatory factor, recent studies have found that it can induce indoleamine 2,3-dioxygenase (IDO), activating the kynurenine (KP) metabolic pathway, leading to the accumulation of neurotoxic metabolites, thereby playing a key role in depressive and anxious behaviors (Hoshi et al., 2009; Kennedy et al., 2017). Our study suggests that this mechanism might also play a significant role in the neuroimmunopathology of PTSD. IL-1β is a classic “alarm” cytokine, prominent in stress-related neuroinflammation, capable of directly affecting synaptic plasticity and neuroendocrine function (Zheng and Zhu, 2005; Prieto et al., 2019). Meanwhile, IL-6, as a core messenger connecting the immune, nervous, and endocrine systems, has elevated levels closely associated with the “negative alterations in cognitions and mood” symptom cluster of PTSD (anhedonia, fatigue) (Kummer et al., 2021). TNF-α has been confirmed to influence central neurotransmitter levels by modulating microglial activity (Prieto et al., 2019; Kraft et al., 2009).

ROC curve analysis in this study showed that IFN-γ, TNF-α, IL-1β, IL-6, and IL-10 all have good predictive efficacy for PTSD (AUC: 0.772–0.820), and the corresponding optimal cut-off values were derived. It should be noted that these proposed thresholds are all from *post-hoc* ROC curve analysis—that is, exploratory analysis based on the existing sample data of this study, with no preset hypotheses during the study design phase. Although this method can fully explore the potential associations in the current data and provide references for preliminary clinical application, it carries a certain risk of bias: on the one hand, the cut-off values are tailored to the sample characteristics of this study, which may lead to overfitting and overestimate its predictive accuracy; on the other hand, the demographic characteristics and clinical characteristics of the single-center sample are relatively concentrated, which may limit the applicability of the cut-off values and result in decreased efficacy when applied to other populations.

### 4.4 Limitations and future directions

This study has several limitations: Firstly, it lacks a control group, making it impossible to fully distinguish the specific differences between the “inflammatory response induced by surgical trauma itself” and the “PTSD-related inflammatory imbalance.” Although confounding factors such as postoperative pain and infection have been controlled through standardized treatment and statistical models, it is still unable to rule out the independent impact of physical stress other than trauma severity on inflammatory factors, which may interfere with the causal interpretation of the results. Secondly, there is a single assessment time point and a lack of longitudinal follow-up. Although 7 days after surgery is a reasonable early time point for assessing inflammation and psychological symptoms, data from only one time point cannot dynamically capture the evolutionary trajectory of inflammatory factors and PTSD, nor can it determine whether the correlation between the two persists in different postoperative stages, potentially missing key dynamic change information. In addition, this study focused on a set of core inflammatory factors and failed to include other important immune indicators such as hs-CRP. It also did not detect downstream metabolites of the IDO-kynurenine pathway potentially mediated by IFN-γ, preventing the mechanism discussion from delving into the molecular pathway level. Finally, the cut-off values of inflammatory factors lack external validation. The predictive thresholds derived through *post-hoc* ROC curve analysis in this study carry the risk of potential overfitting bias, and their stability and applicability have not been verified in independent cohorts, which may lead to errors when directly applied to clinical screening.

Future studies can adopt a multi-center, prospective cohort design, conduct dynamic monitoring at multiple postoperative time points, and integrate more comprehensive detection of immune indicators and neurotoxic metabolites to clarify the causal role of inflammation in the occurrence and development of PTSD and the specific biological pathways. Crucially, it is necessary to verify and calibrate the inflammatory factor cut-off values derived in this study through large-sample, multi-center independent external cohorts, while optimizing the screening process in combination with clinical practice, to transform them into reliable clinical early warning tools.

## 5 Conclusion

The results of this study indicate that post-traumatic stress disorder can manifest early in patients following digit replantation, with its severity influenced by factors such as female gender, lower education level, trauma severity, and co-morbid anxiety and depression. More importantly, PTSD symptoms were positively correlated with serum levels of pro-inflammatory factors (IFN-γ, TNF-α, IL-1β, IL-6) and negatively correlated with the level of the anti-inflammatory factor IL-10. This association was specifically present in the patient group with PTSD, revealing the core role of a pro-inflammatory/anti-inflammatory immune imbalance in the pathological state of PTSD. This study provides, for the first time in the specific trauma model of digit replantation, empirical evidence supporting the “trauma-neuro-immune” vicious cycle hypothesis. It suggests the potential of inflammatory factors serving as early warning biomarkers, laying a theoretical foundation for future development of immune mechanism-based precision prevention and control strategies for PTSD.

## Data Availability

The raw data supporting the conclusions of this article will be made available by the authors, without undue reservation.
